# UV degradation of the interface between perovskites and the electron transport layer†

**DOI:** 10.1039/c9ra10960a

**Published:** 2020-03-20

**Authors:** Ranran Liu, Li Wang, Yingping Fan, Zhipeng Li, Shuping Pang

**Affiliations:** Qingdao University of Science and Technology, College of Materials Science and Engineering Qingdao 266042 P. R. China liwang718@qust.edu.cn; Qingdao Institute of Bioenergy and Bioprocess Technology, Chinese Academy of Sciences Qingdao 266101 P. R. China

## Abstract

The stability of the perovskite/electron transport layer (ETL) interface is critical for perovskite solar cells due to the presence of ultraviolet (UV) light in the solar spectrum. Herein, we have studied the decomposition process and performance evolution of the perovskite layer in contact with different ETLs under strong ultraviolet irradiation. The normally used SnO_2_ layer has lower photocatalytic activity in comparison with the TiO_2_ layer, but the perovskite/SnO_2_ interface is still severely decomposed along with the formation of hole structures. Such UV light-induced decomposition, on the one hand, leads to the decomposition of the perovskite phase into PbI_2_ and more seriously, the formed hole structure significantly limits the carrier injection at the interface owing to the separation of the perovskite active layer from ETLs. Under the same conditions, the perovskite/PCBM interface is very stable and maintains a highly efficient carrier injection. There is no significant efficiency degradation of the encapsulated PCBM-based devices measured outdoors for about three months.

## Abstract 2

Using SnO_2_ as the ETL in perovskite solar cells can degrade the interface and cause device performance degradation under UV light.

## Graphical abstract

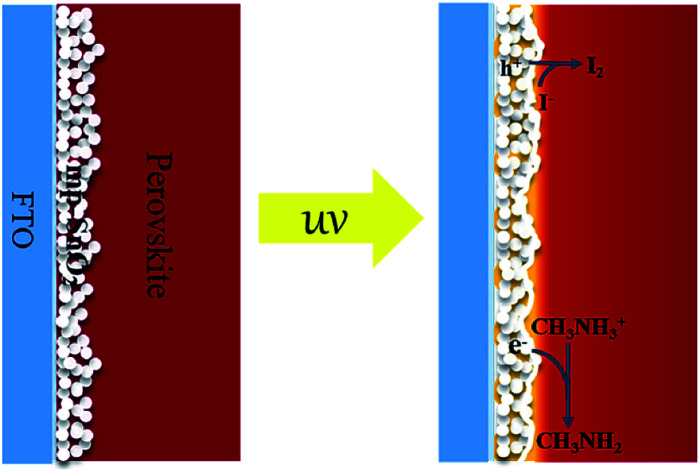

## Introduction

The power conversion efficiency (PCE) of perovskite solar cells (PSCs) has risen from 3.8% up to 25.2% in a decade.^1,2^ However, a key issue for the commercialization of PSCs is to increase their long-term stability.^3–5^ Compared with the humidity and thermal stability, the stability under light irradiation is more challenging.^6–8^ The mechanism of light-induced degradation is virtually distinctive under different atmospheres.^9–15^ It has been found that when PSCs are exposed to light and oxygen, O_2_^−^ is generated by the reaction of photoelectrons and O_2_ and it can react with CH_3_NH_3_^+^ from the perovskite crystals; finally, the CH_3_NH_3_PbI_3_ layers degrade rapidly to CH_3_NH_2_, PbI_2_, and I_2_.^9,10^ In an atmosphere containing light and moisture, water molecules normally interact with I^−^ in the perovskite crystals to cause CH_3_NH_3_PbI_3_ to degrade to CH_3_NH_2_, PbI_2_, and HI.^11^ When PSCs are encapsulated in nitrogen, the deep surface trap sites (Ti^3+^) at the surface of TiO_2_ can trap the photoelectrons and increase the recombination of electrons and holes under illumination.^12,13^ Generally, the poor light stability of PSCs is because of the presence of ultraviolet (UV) light in the solar spectrum. Under UV light, the traditionally used mesoporous (mp) TiO_2_ electron transport layer with high photocatalytic properties can be excited to generate electron–hole pairs.^16,17^ Subsequently, the photoholes can extract electrons from I^−^ and the photoelectrons can deprotonate CH_3_NH_3_^+^ at the perovskite/mp-TiO_2_ interface, ultimately leading to the photo-degradation of perovskite crystals into CH_3_NH_2_ and HI gases and PbI_2_.^18^ To remove the impact of mp-TiO_2_ on the device stability, the ETL upon substitution of mp-TiO_2_ with mp-SnO_2_ has been employed and studied.^19–22^ After exposure to 1 sun light for 10 h, the stability is improved by about 11% at the maximum power output in comparison with that for the mp-TiO_2_-based devices.^23^ It was also found that the surface of TiO_2_ passivated by SnO_2_ and the double-layer structure of amorphous SnO_2_-coated TiO_2_ can both retard the photocatalytic activity of TiO_2_ and improve the UV stability of the devices.^24,25^ In addition to SnO_2_, the inverted device based on organic PCBM ETL has great stability under UV exposure.^26^ It is well-known that the stability of inverted PSCs with PCBM ETL is superior to that of the normal devices with TiO_2_ or SnO_2_ as the ETLs. The above-mentioned evidence strongly indicates that the interface of the perovskite/electron transport layer (ETL) is critical for the light stability of PSCs. However, the interface decomposition process of the perovskite layer in contact with different ETLs under UV irradiation has not been systematically studied. Considering that moisture and oxygen will be isolated by encapsulation, the study of the UV light stability in an inert environment will be of more practical significance and wider applicability.

Herein, we employed high-intensity UV light to study the stability of the perovskite films in contact with different ETLs in a nitrogen-filled glovebox. It has been found that the low light stability is because of the fast interface decomposition, which leads to the decomposition of the perovskite phase into PbI_2_ and the separation of the perovskite active layer from ETL. The PCE of the device based on mp-SnO_2_ indeed shows slower degradation than that based on mp-TiO_2_ ETL, while the PCBM-based device shows no significant morphology, composition and PCE degradation under the same testing period.

## Results and discussion

**Fig. 1:**
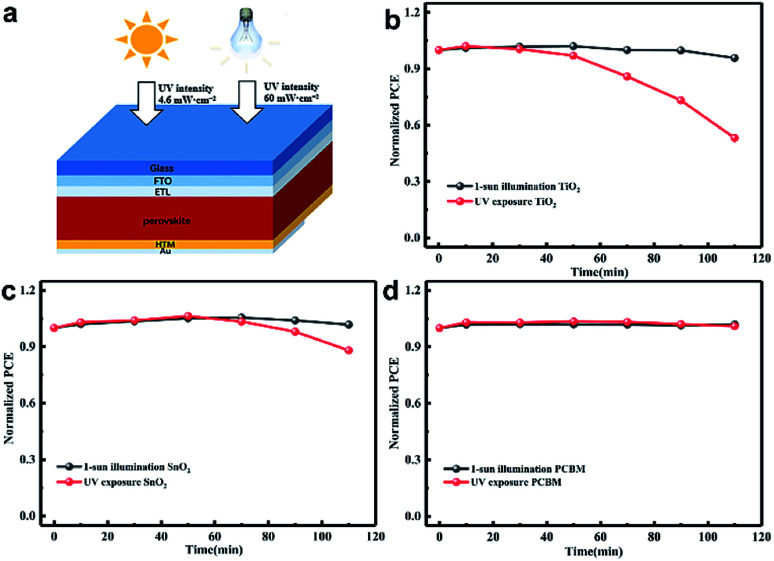
(a) Schematic representation of perovskite solar cells for light irradiation testing; the normalized PCEs of (b) mp-TiO_2_, (c) mp-SnO_2_ and (d) PCBM-based devices with the increase of 1 sun illumination and UV exposure time. All the devices were treated in a nitrogen-filled glovebox and measured outside.

Fig. 1(a) shows the structure diagram of the device under 1 sun AM 1.5 G (100 mW cm^−2^) intensity or strong UV light (60 mW cm^−2^) exposure. The unencapsulated devices were illuminated in a nitrogen-filled glovebox to eliminate the effect of moisture and oxygen. The UV intensity in the AM 1.5 G solar spectrum was approximately 4.6 mW cm^−2^ at wavelengths of less than 400 nm.^27^ We employed 365 nm UV illumination in this experiment and its intensity was 60 mW cm^−2^, which is about 13 times higher than that of 1 sun. The light was incident from the FTO side, and the UV intensity passing through the FTO dropped to about 60% of the original value as shown in Fig. S1.† The stability results of the normal device with FTO/bl-TiO_2_/mp-TiO_2_/perovskite/Spiro-OMeTAD/Au structure are shown in Fig. 1(b) with 1 sun illumination and UV exposure, respectively. The PCE was almost identical before and after 1 sun illumination for 110 min but it significantly declined during the UV exposure. The PCE decreased after UV treatment for 30 min and about 50% degradation of the PCE occurred after 110 min. The *J*–*V* characteristics of the fresh and UV aged devices are shown in Fig. S2.† As shown in Fig. 1(c), the use of mp-SnO_2_ as the ETL provided significant improvement in light stability as compared with mp-TiO_2_ based devices. A distinct decline in PCE occurred under UV exposure for 50 min and the PCE decreased by only approximately 10% of the original value after 110 min of UV treatment. More interestingly, the inverted devices with PCBM as the ETL (FTO/NiO_*x*_/perovskite/PCBM/Au) exhibited more stable performances than the two types of devices mentioned above. The PCE showed no attenuation after sunlight or UV treatment according to Fig. 1(d). This indicates that UV illumination greatly affected the stability of the metal oxide-based device and ETL is strongly related to long-term photo-stability, but it remains unclear how ETL works on decreasing the PCE of the devices under UV illumination.

**Fig. 2:**
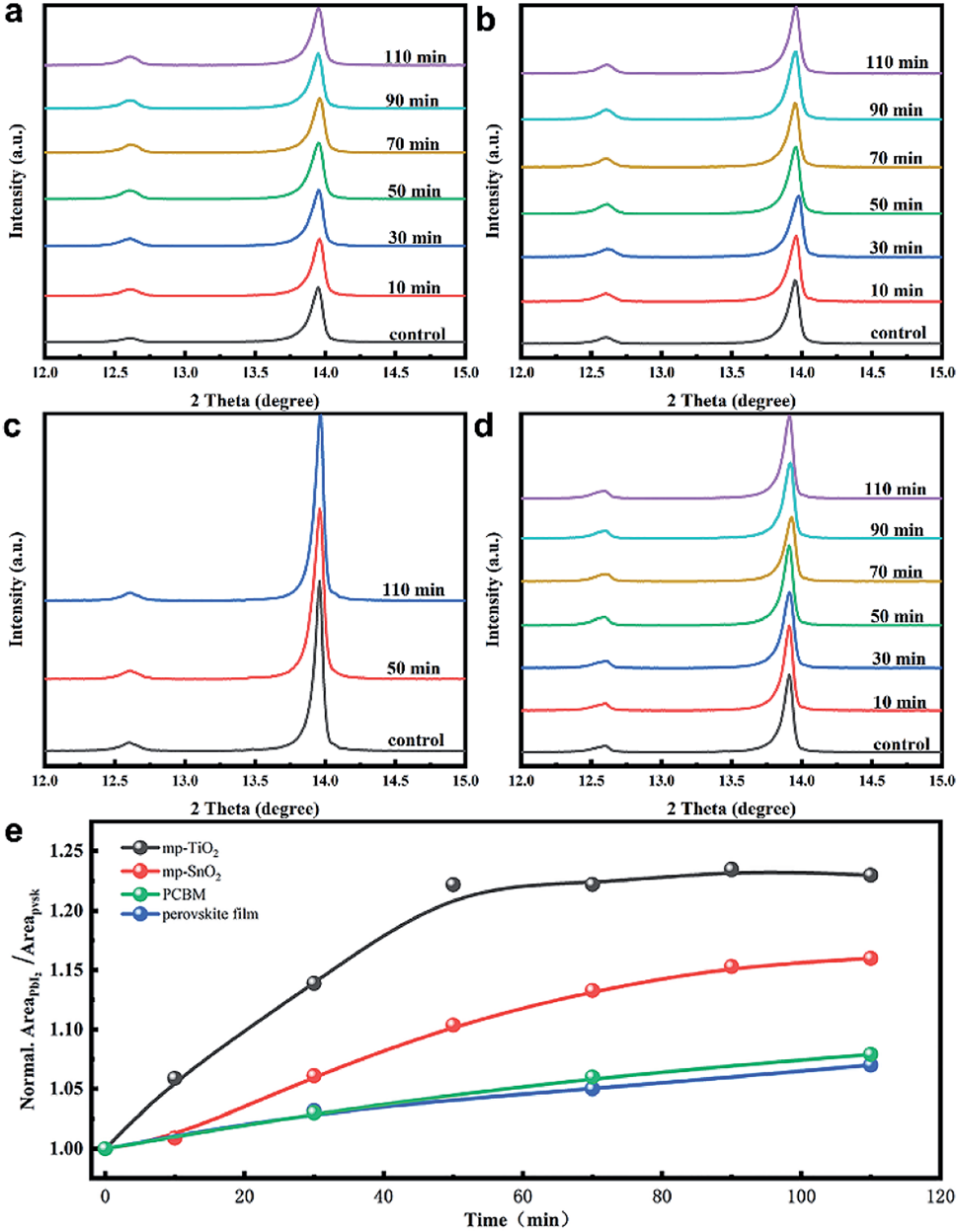
X-ray diffraction patterns of perovskite films in contact with (a) mp-TiO_2_, (b) mp-SnO_2_, (c) PCBM, and (d) glass with increasing UV exposure time. (e) The evolution of the diffraction peak area ratio between (001) of PbI_2_ (12.6°) and (110) of perovskite (13.9°) with different UV irradiation times.

**Fig. 3:**
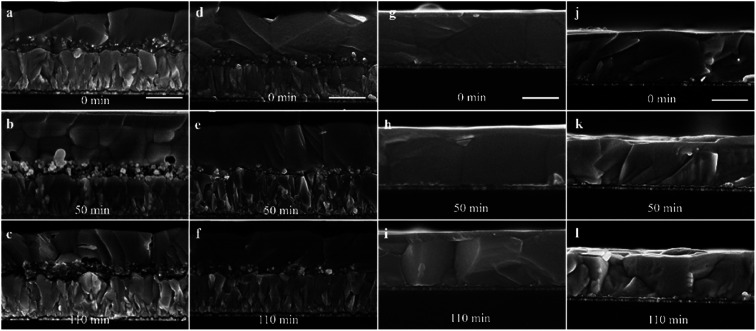
The cross-sectional SEM images of perovskite films based on (a–c) mp-TiO_2_, (d–f) mp-SnO_2_, (g–i) PCBM and (j–l) pure glass after 0, 50, 110 min UV exposure, respectively; the scale bar is 500 nm.

In order to understand the effects of UV treatment on the perovskite film, the composition evolution of the perovskite films was traced with X-ray diffraction (XRD) measurement as presented in Fig. 2. It should be noted that all the aged perovskite films in this study were irradiated from the FTO side. PbI_2_ diffraction peaks at 12.6° were observed for the fresh perovskite films on all substrates (mp-TiO_2_, mp-SnO_2_, glass), which is consistent with the results reported in literature.^28^ The enhanced intensity of the PbI_2_ diffraction peak along with UV treatment indicated that the PbI_2_ content in perovskite layers increased, which revealed the decomposition of the perovskite crystals on mp-TiO_2_ (Fig. 2(a)) and mp-SnO_2_ (Fig. 2(b)) substrates under UV exposure. After 110 min of UV treatment, the perovskite layers on the glass substrate with PCBM (Fig. 2(c)) and without PCBM as a reference (Fig. 2(d)) showed that the intensity of PbI_2_ changed negligibly, which suggests that the outstanding UV stability of the perovskite layers and the PCBM ETL has no effect on the UV stability of the perovskite layers. The normalized peak area ratio between (001) of PbI_2_ (12.6°) and (110) of perovskite (13.9°) with the increase in UV exposure time is shown in Fig. 2(e). The perovskite film on the glass substrate was stable, and their ratios increased slightly after UV treatment. However, the peak area ratio in the perovskite film on the mp-TiO_2_ substrate increased rapidly, reaching 1.23 after 50 min and then became stable. This is because the TiO_2_/perovskite interface was badly degraded, the perovskite was separated from TiO_2_ as shown in Fig. 3(c), and the degradation process could not occur further due to the indirect contact with TiO_2_. The mp-SnO_2_ based perovskite film also showed an increasing trend but not as much as the improvement observed for the mp-TiO_2_ and the ratio was up to 1.16 after 110 min of UV treatment. According to the literature, although moderate PbI_2_ content can passivate the surface or grain boundary defects and enhance the device performance, excess PbI_2_ left in the perovskite can influence absorption and cause poor performance.^29–31^ It was surprising that even though the diffraction peak of PbI_2_ was stronger than that of perovskite, the PCE decreased slightly.^32^ In this experiment, the PCE of the device based on mp-TiO_2_ dramatically attenuated to approximately 50% after exposure to UV for 110 min. Even though the content of PbI_2_ in the perovskite film was enhanced, it was far below the content reported in the literature (Fig. 1(b)). This confirms that the increased PbI_2_ content in perovskite film is not the factor that directly caused the decline in the PCE.

To further probe the effect of UV irradiation on the stability of the perovskite film in contact with different ETLs, the cross-sectional Scanning Electron Microscopy (SEM) images in Fig. 3 were obtained to compare the morphology evolution of the perovskite film before and after UV irradiation exposure. The morphology of the perovskite film without ETL was constant before and after illumination, indicating the excellent stability of the perovskite film during this treatment process (Fig. 3(j–l)). However, the morphology of the mp-TiO_2_/perovskite interface changed evidently and the dense perovskite layer in the mp-TiO_2_ was converted into a loose structure with pinholes after UV treatment for 50 min (Fig. 3(b)). With the increase in the UV treatment time, the interface decomposition was more serious, resulting in interface separation as shown in Fig. 3(c). This phenomenon is attributed to the strong photocatalytic behavior of mp-TiO_2_, and the photoholes in the valence band can extract electrons from I^−^ to produce I_2_, which decomposes the perovskite crystal. Simultaneously, the photoelectrons in the conduction band can break down the perovskite by first deprotonating CH_3_NH_3_^+^, ultimately resulting in the formation of CH_3_NH_2_ gas and PbI_2_.^12^ The possible reaction at the interface can be described by the following equation:^18^CH_3_NH_3_PbI_3_ → PbI_2_ + CH_3_NH_2_ (aq) + HI (aq)

The FA-based perovskite film is similar to this degradation process. SnO_2_ is relatively inert in photo-catalysis as reported in the literature and was used to replace or passivate mp-TiO_2_ to improve UV stability.^23–25^ However, we still found the poor contact and pinhole structures at the interface of the mp-SnO_2_/perovskite after UV treatment as presented in Fig. 3(e and f). The interface of the planar SnO_2_/perovskite showed more severe degradation for the same time (Fig. S6†), and the stability of the planar structure was poorer than that of the mesoporous structure, which is consistent with reported results.^33,34^ It can be inferred that SnO_2_ also has a photocatalytic effect on the decomposition of perovskite at the mp-SnO_2_/perovskite interface under UV exposure. Fig. 3(h and i) shows the stable perovskite/PCBM interface after different UV treatment times. Note that the UV illumination from the PCBM side also shows a similar effect to illumination from the FTO side (Fig. S3†).

It is worth pointing out that the environment has a great impact on the decomposition behavior. We have also placed the perovskite films based on mp-TiO_2_ or mp-SnO_2_ in an open environment with humidity of about 40% for the UV aging experiment. The results show that the degradation at the ETL/perovskite interface is more serious than that in a nitrogen-filled glovebox (Fig. S7†). Especially at the mp-TiO_2_/perovskite interface, the dense perovskite was completely destroyed to form a porous structure. The intensity of the PbI_2_ diffraction peak increased significantly (Fig. S5†) because of the presence of oxygen. Under UV illumination, TiO_2_ produced photoelectrons in the conduction band, which were subsequently trapped by O_2_ in the air to form the superoxide O_2_˙^−^ and thus caused the decomposition of perovskite by first deprotonating the organic cation.^24^
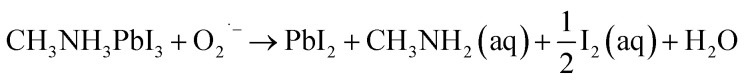


**Fig. 4:**
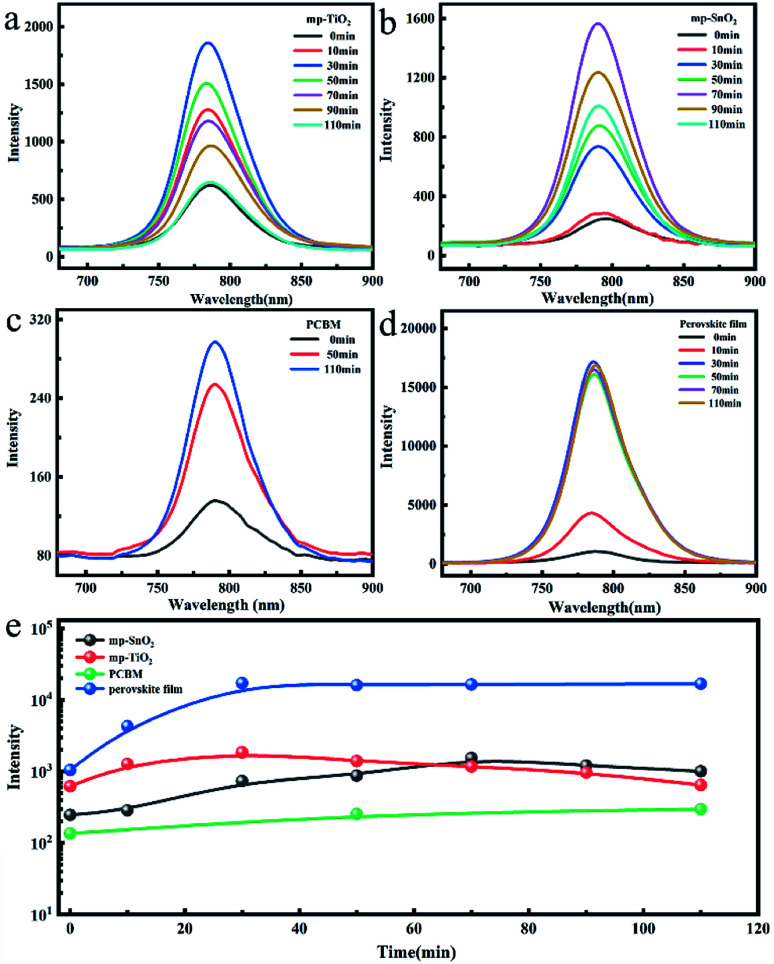
The steady-state photoluminescence (PL) spectra of perovskite films based on (a) mp-TiO_2_, (b) mp-SnO_2_, (c) PCBM, and (d) glass with the increase in the UV exposure time. (e) The intensity of the above perovskite film after different UV irradiation times. The 450 nm laser was incident from the glass surface.

The change in the photoluminescence (PL) performance along with UV treatment was also studied to evaluate the carrier injection efficiency at the interface as shown in Fig. 4. The PL intensity of the perovskite film without ETL increased continuously to 16 times after 30 minutes of UV exposure and then remained stable within the subsequent illumination time as shown in Fig. 4(d and e). This may be attributed to the effective UV-treatment passivation at the surface and grain boundary defects and the suppressed non-radiative recombination, resulting in a lower trap density and enhanced electron extraction.^26^ The PL intensity of the perovskite film deposited on mp-TiO_2_ ETL increased to about 3 times during the first 30 minutes of UV treatment and then constantly decreased as presented in Fig. 4(a and e).

With the increase in UV exposure time, the perovskite film was continuously degraded, while holes formed at the mp-TiO_2_/perovskite interface due to the photocatalysis of mp-TiO_2_ (Fig. 3(b and c)). The degraded and separate interface failed to inject electrons, which also caused the enhancement in the PL intensity, but the continuous decomposition of perovskite crystals eventually led to the rapid decline in the PL intensity. Analogously, the intensity of the mp-SnO_2_-based perovskite film exhibited the same trend. The time to reach the maximum was longer and the intensity was up to about 6 times after 70 min UV treatment (Fig. 4(b and e)).

We also analyzed the change in the PL intensity of the laser incident from the perovskite layer side as indicated in Fig. S6.† Compared with the laser from the glass side, the PL intensity of the perovskite films deposited on mp-TiO_2_ or mp-SnO_2_ also increased first and then decreased with the extension of the UV treatment time. This confirmed that mp-SnO_2_ and mp-TiO_2_ have similar photocatalytic effects, causing the instability of the perovskite phase at the contact position. Fig. 4(c) indicates that the PL intensity of the perovskite film based on PCBM is relatively weak because of the efficient electron injection efficiency at the perovskite/PCBM interface. It shows that the PL intensity constantly increased to about 2 times after UV treatment for 110 min. The changing trend of PL intensity is consistent with that of the perovskite film on the glass substrate. Therefore, we think that the interface between perovskite and PCBM is stable and the carrier injection efficiency cannot be weakened after this period of UV exposure.

**Fig. 5:**
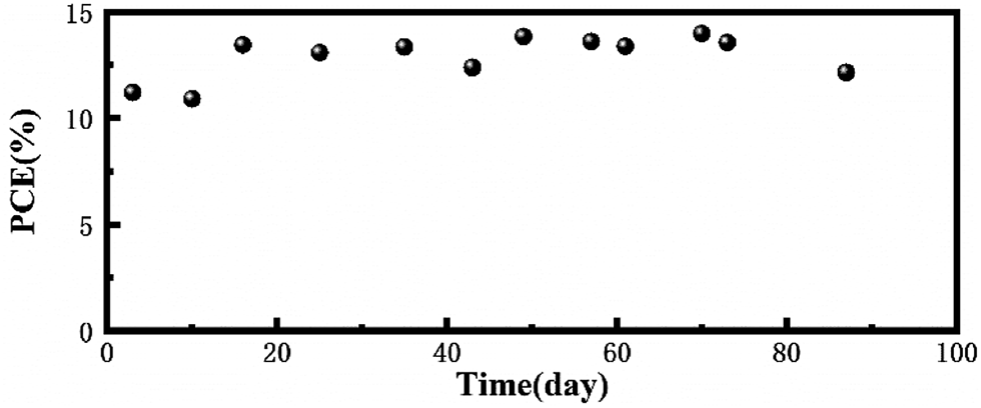
Stability testing of the encapsulated PCBM-based device under continuous light illumination in outdoor conditions.

To prove the long-term light stability of the PCBM based device, we fabricated an inverted device with FTO/NiO_*x*_/Perovskite/PCBM/ITO structure and all the devices were encapsulated under a nitrogen atmosphere and then placed outdoors in working condition. The area of the device was 0.196 cm^2^ and this measurement was performed under 1 sun AM 1.5 G (100 mW cm^−2^) intensity. The PCE degradation of the encapsulated devices was negligible within about three months, which revealed that the PCBM-based device has excellent long-term light stability (Fig. 5).

## Conclusions

In summary, it has been demonstrated that the interface between the perovskite and the ETL is extremely important for PSCs. Under UV light, the perovskite/mp-TiO_2_ interface showed more serious decomposition behavior. Although SnO_2_ is considered as a stable ETL for perovskite solar cells, the mp-SnO_2_/perovskite interface also decomposed along with the formation of hole structures and increased PbI_2_ content. The formed hole structure badly limits the carrier injection at the interface owing to the separation of the perovskite active layer from ETLs and thus, the device performance sharply decreased. Gratifyingly, the perovskite/PCBM interface maintained superior stability and efficient carrier injection after the same UV-treatment. The encapsulated PCBM based devices showed no significant efficiency degradation after being tested outdoors for three months.

## Experimental

### Materials

All reagents and solvents were used without further purification. Methylamine iodine (MAI), methylammonium bromide (MABr), formamidinium iodide (FAI) and methylammonium chloride (MACl) were purchased from Xi'an Polymer Light Technology Corp. Other reagents were purchased from Sigma-Aldrich. TiO_2_ gel was synthesized in the lab as previously reported.^35^

### The preparation of SnO_2_ nanoparticles and paste

The SnO_2_ nanoparticles (NPs) were synthesized by the hydrothermal method as mentioned in the literature.^36–40^ Specifically, 0.1 ml of anhydrous SnCl_4_ was dissolved in 2.5 ml anhydrous alcohol under stirring. Aqueous TMAH (25 wt% in water) was then dripped into that solution until the pH was around 11 and the resulting white floc disappeared. After that, the solution was transferred to a Teflon-lined stainless autoclave (20 ml) and heated at 220 °C for 12 h. The precipitate was dispersed in deionized water, centrifuged at 10 000 rpm for 10 min and washed at least 3 times. Finally, the SnO_2_ NPs were dissolved in trifluoroethanol and the solution concentration was 30 mg ml^−1^. Fig. S8† shows that the size of the SnO_2_ NPs is about 20 nm. The SnO_2_ paste was prepared by adding 5 ml of SnO_2_ NPs solution to 20 g of terpineol and 15 g of ethylcellulose. This process is described elsewhere.^19^ The paste was fully ground in an agate mortar.

### Perovskite film and device fabrication

Fluorine-doped tin oxide glass (FTO) substrates (14 Ω cm^−2^, 20 × 15 mm^2^) were partly etched with Zn powder and 1 M HCl. The etched substrates were cleaned with acetone, isopropanol with KOH, and deionized water for 15 min and then dried with clean dry air. To prepare TiO_2_ ETL, a thick (20 nm), compact TiO_2_ hole-blocking layer was deposited on the FTO substrate by atomic layer deposition (ALD). Subsequently, a 200 nm thick mesoporous TiO_2_ layer was prepared on the TiO_2_ compact layer by spin coating a diluted commercial TiO_2_ gel (1 : 6 with ethanol by weight) at 4000r for 30 s and then annealing at 500 °C for 30 min in air. To prepare SnO_2_ ETL, a thick (20 nm), compact SnO_2_ layer was deposited on the FTO substrate by spin-coating commercial SnO_2_ particles (diluted 1 : 6 with deionized water by volume) at 3000 rpm for 30 s, followed by annealing at 200 °C for 30 min in air. The thin (∼150 nm) mesoporous SnO_2_ layers were obtained by spin-coating the SnO_2_ paste diluted in trifluoroethanol (1 : 1, weight ratio) at 3000 rpm for 30 s onto the compact SnO_2_ substrates, followed by annealing at 500 °C for 30 min in air as shown in Fig. S8.† To prepare PCBM ETL, a thick (100 nm) PCBM layer was deposited on the perovskite layer by spin-coating PCBM solution at 3000 rpm for 30 s. The perovskite layer was prepared as reported in the literature.^29^ Briefly, the precursors were prepared by mixing PbI_2_ : FAI : MABr : MACl (1.55 : 1.35 : 0.2 : 0.3 molar ratio) in a mixture solvent of DMSO and DMF (v : v, 9 : 1). Then, the precursor solution was spin-coated onto different substrates at a speed of 2000 rpm for 60 s, and 300 μL of chlorobenzene was dropped onto the surface of perovskite films as the anti-solvent at 5000 rpm for 30 s. The films were then annealed at 150 °C for 20 min in ambient air (30–40% humidity). The Spiro-OMeTAD hole-transporting layer (HTM) was prepared by dissolving 72.3 mg of Spiro-OMeTAD in 1 ml of chlorobenzene, to which 28.8 μL of 4-*tert*-butylpyridine (96%, Aldrich-Sigma) and 17.5 μL of lithium bis (trifluoro-methanesulfonyl) imide (Li-TSFI, Aldrich-Sigma) solution (520 mg Li-TSFI (98%) in 1 ml acetonitrile (99.8%, Aldrich-Sigma)) were added. After complete dissolution, 30 μL of Spiro-OMeTAD solution was deposited by spin-coating at 3000 rpm for 30 s. The 30 nm thick NiO_*x*_ HTM was prepared by spray pyrolysis as mentioned in the literature.^41^ Finally, 100 nm thick Au electrodes were thermally evaporated under vacuum to complete the PSC fabrication.

### Material and device characterization

XRD spectra were obtained using a Bruker-AXS Micro diffractometer (D8 ADVANCE) with Cu Kα radiation (1.5406 Å). The optical absorbance spectra were obtained on a UV-vis/NIR spectrophotometer (U-4100, Hitachi). Top-view, cross-sectional SEM images were obtained with a field-emission scanning electron microscope (S-4800, Hitachi). The TEM image was obtained with a transmission electron microscope (JEM-1011). Steady-state photoluminescence (PL) spectra were recorded on a Perkin LS-55 fluorescence spectrometer, with excitation at 450 nm. *I*–*V* curves of the fabricated PSCs with different scanning directions were measured using a 2400 Sourcemeter (Keithley, USA) under simulated one sun AM 1.5 G 100 mW cm^−2^ intensity (Oriel Sol3A Class AAA, Newport, USA). The typical active area of the PSCs was 0.09 cm^2^, defined by a metal mask. The intensity of the 1 sun AM 1.5 G illumination was calibrated using a Si-reference cell certified by the National Renewable Energy Laboratory.

## Conflicts of interest

There are no conflicts to declare.

## Supplementary Material

RA-010-C9RA10960A-s001
