# Supplementary material for: UV degradation of the interface between perovskites and the electron transport layer
Source: RSC Adv. 2020 Mar 20;10(20):11551–6. doi: 10.1039/c9ra10960a (PMC9050615; doi:10.1039/c9ra10960a)
Supplement: RA-010-C9RA10960A-s001 [file RA-010-C9RA10960A-s001.pdf]

Electronic Supplementary Material (ESI) for RSC Advances.
This journal is © The Royal Society of Chemistry 2020

Supporting Information

UV Degradation of the Interface between Perovskite and Electron Transport Layer

Ranran Liu,[ab] Li Wang,[*a] Yingping Fan,[ab] Zhipeng Li,[b] and Shuping Pang[b]

[a] Qingdao University of Science and Technology, College of Materials Science and Engineering Qingdao 266042, P.R. China. Email: liwang718@qust.edu.cn

[b] Qingdao Institute of Bioenergy and Bioprocess Technology, Chinese Academy of Sciences, Qingdao 266101, P.R. China.

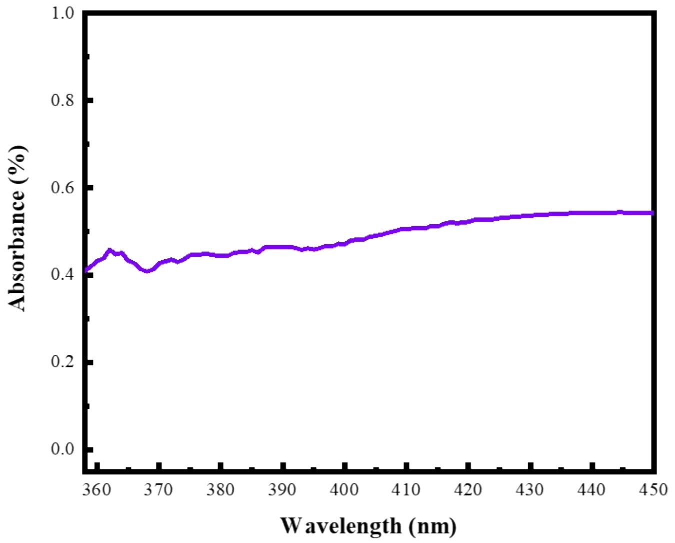

Figure S1 The optical absorbance spectra of FTO coated glass were measured by UV-vis/NIR spectrophotometer and its absorption coefficient at 365nm is 0.42. About 60 percent of ultraviolet light intensity can be transmitted and reach ETL.

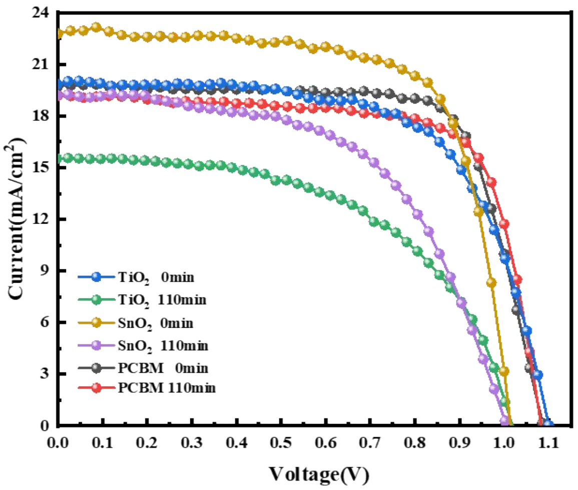

Figure S2 The typical *J–V* curves of the devices based on different ETLs before and after UV treatment.

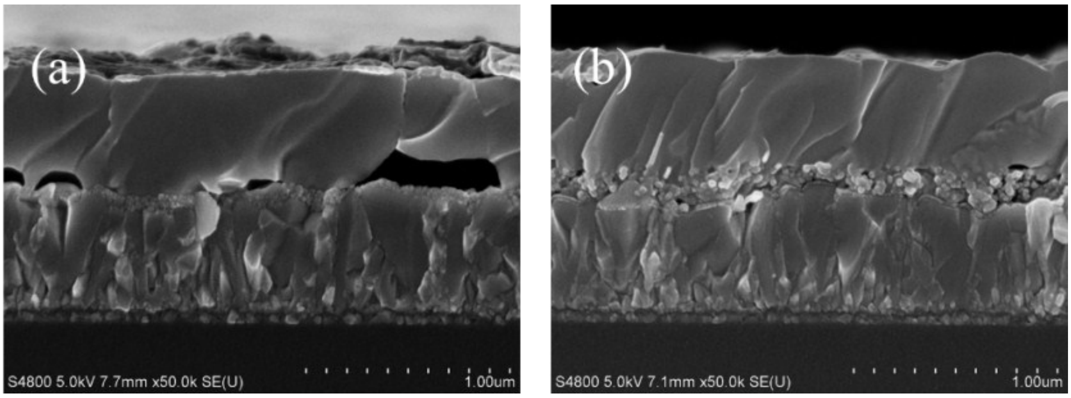

Figure S3 The cross-sectional SEM images of perovskite films based on (a) planar SnO_2, (b) mp-SnO_2 after 110 min UV exposure, respectively.

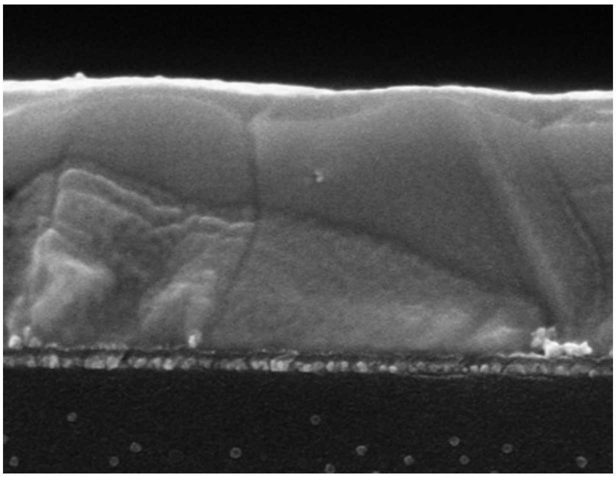

Figure S4 The cross-sectional SEM images of perovskite films based on PCBM after 110min UV treatment and the UV illumination was from the PCBM side. The morphology of PCBM/perovskite interface is stable which shows a similar effect as illumination from FTO side.

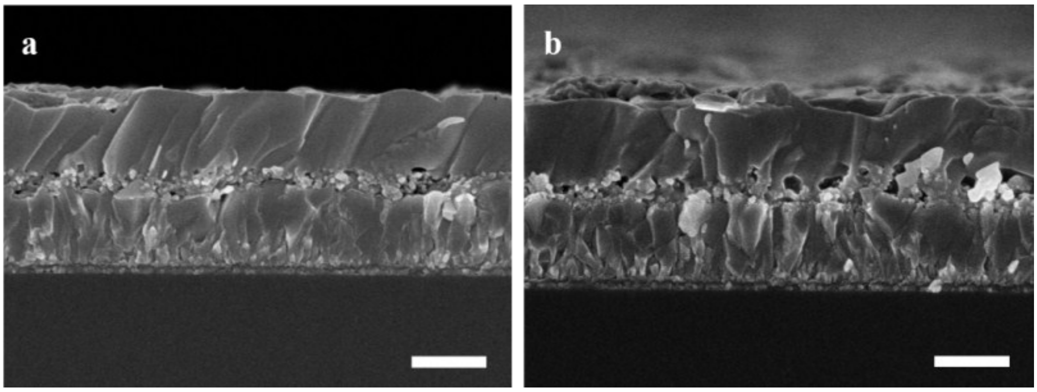

Figure S5 The cross-sectional SEM images of perovskite layers deposited on (a) mp-SnO_2, (b) mp-TiO_2 after 110min UV illumination in an open environment with a humidity of about 40%. The interface of mp-SnO_2/ perovskite or mp-TiO_2/perovskite is more serious than that in a nitrogen glove box.

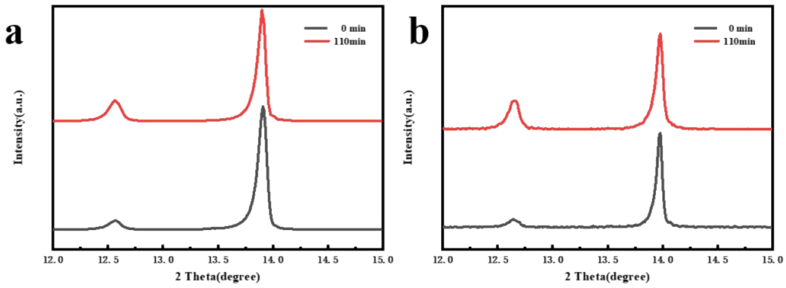

Figure S6 X-ray diffraction patterns of (a) mp-SnO_2, (b) mp-TiO_2 based perovskite film after 110min UV irradiation in an open environment with a humidity of about 40%. The intensity of PbI_2 diffraction peak increased significantly which indicates the stronger photocatalysis of TiO_2 and SnO_2 than that in N_2 atmosphere.

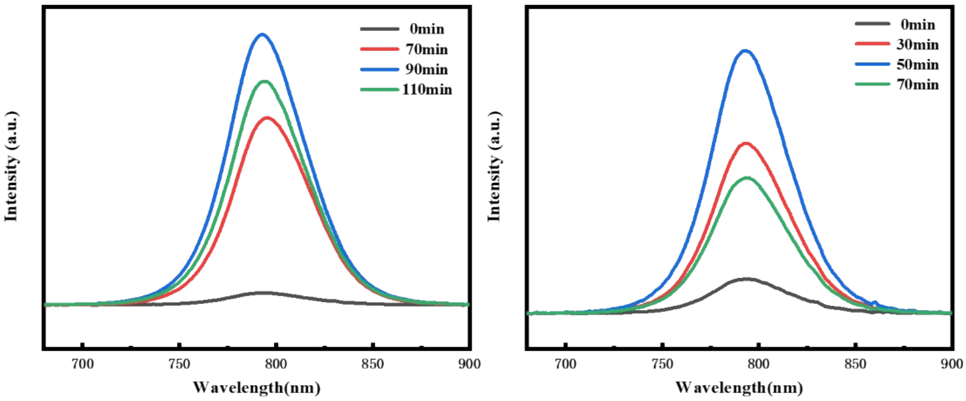

Figure S7 Steady-state photoluminescence (PL) spectra of perovskite film based on (a) mp-TiO_2, (b) mp-SnO_2. The 450nm laser is incident from the perovskite film surface. Compared with the laser from the glass side, the PL intensity of perovskite films deposited on mp-TiO_2 or mp-SnO_2 also shows the trend of increasing first and then decreasing with the extension of UV treatment time.

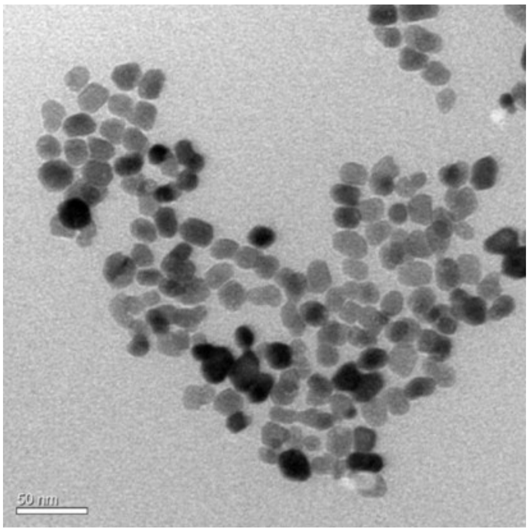

Figure S8 TEM image of SnO_2 nanoparticles (NPs). The grain size of the nanoparticles is approximately 20 nm and the SnO_2 NPs were uniformly dispersed and there was no agglomeration.

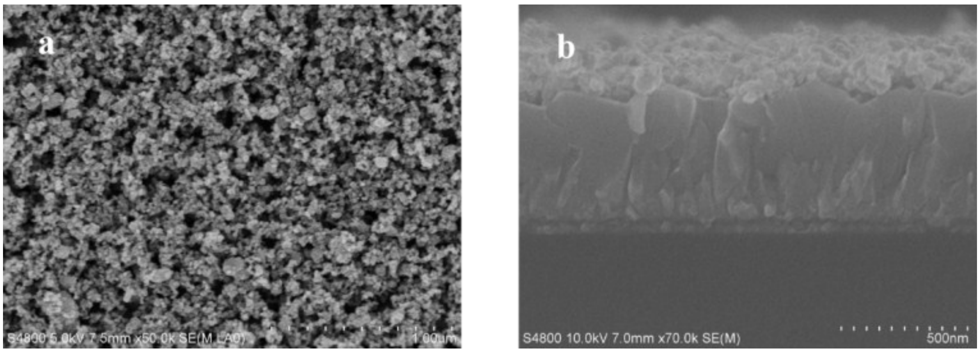

Figure S9 (a) Surface SEM image of SnO_2 nanoparticles. (b) Cross-sectional SEM images of SnO_2 NPs deposited on FTO. The thickness of mesoporous (mp) SnO_2 layer is about 150 nm.
